# The Macrophage (2nd Edn)

**DOI:** 10.1038/sj.bjc.6601103

**Published:** 2003-07-15

**Authors:** M J Embleton

**Affiliations:** 1 University of Manchester, UK

The second edition of The Macrophage comes at a time of resurgence of interest in innate immunity. The innate immune system depends upon a range of effector cells with genetically determined receptors recognising conserved molecular structures expressed by a wide variety of potential invading organisms, allowing it to distinguish between self and non-self. It thus differs from the adaptive immune system comprising B and T lymphocytes, in that it does not rely upon clonal selection of effector cells with unique antigen-specific receptors provided by rearrangement of multiple gene segments. This means that the innate system is capable of relatively rapid reactions, limited only by the time taken for cytokine cascades to take effect. Foremost among the cell types involved in the innate immune response are macrophages. Macrophages have a range of important functions in relation to inflammatory responses against foreign organisms, including phagocytosis of smaller cells such as bacteria, direct or antibody-directed killing of these and other organisms, and interaction with the adaptive immune system particularly in the area of antigen processing. With regard to cancer, macrophages form a significant proportion of the total cell population in the vast majority of tumour tissues and there is abundant evidence that under some conditions, they are capable of cytotoxic or cytostatic activity against malignant cells. However, there is a down side to all of this. Macrophage activity can also be destructive to the host, as, for example, its involvement in the development of atheromatous plaques and rheumatoid arthritis, and in cancer macrophages very often promote the survival and spread of tumour cells rather than inhibit them, depending on the local cellular and cytokine environment. This ambivalence was amply portrayed in the first edition of The Macrophage and is an unchanging consequence of the complex biology of macrophages. The complexity of tumour biology and the heterogeneity of tumours in general only serve to compound the problem for oncologists.

The second edition of The Macrophage is a well-presented volume running to almost 650 pages. It contains 16 chapters by acknowledged experts, and the topics range from macrophage biology at both cellular and molecular levels, through interactions with different target cells and organisms and the role of macrophages in various pathological conditions in the host, to mathematical models and gene therapy. The chapters perhaps most likely to be of direct interest to oncologists are those entitled The Biology of the Macrophage, Macrophages as Antigen-Presenting Cells: Relationship to Dendritic Cells and Use in Vaccination Studies, Macrophage–Virus Interactions, Macrophages in Tumour Biology and Macrophages in Gene Therapy: Cellular Targets and Gene Delivery Vehicles. However, this is certainly not to decry any of the other chapters in the book. Covering every aspect of both pathological and normal functions of macrophages as it does, this book provides a wealth of detailed and up-to-date information relevant to anyone with more than a passing interest in macrophages, immunology or cellular interactions in general. A major feature repeated throughout the book is the vast increase in knowledge of interactions between macrophages and other cell types, particularly those involving cytokine/lymphokine interactions, and of associated cell signalling. Interactions involved in the activation and regulation of macrophages are still potentially confusing because of the multitude of factors involved and often the multiplicity of effects of a given factor or conversely their redundancy, but with further understanding they could hold the key to managing the functions and activities of macrophages in a more controlled way. Despite the rather pessimistic caveat about macrophages in the above paragraph, many of the authors have also highlighted other areas of promise for the role of macrophages in the alleviation of a variety of diseases, including cancer. Past attempts at cancer immunotherapy by exploiting the activation of effector cells (predominantly macrophages) by diverse natural or synthetic adjuvants were mostly ineffectual, apart from isolated examples such as the treatment of bladder cancer with BCG . However, there is now clear hope of future treatments for many types of cancer based on the use of macrophages and dendritic cells as antigen-presenting cells to activate the adaptive immune response for active T-cell immunotherapy and in some cases as prophylactic vaccines. There is also great potential for gene therapy in which macrophages could play a role as both targets for gene manipulation and as vehicles for targeted delivery of recombinant genes to particular tissues. These aspects offer promise for the treatment of many other diseases besides cancer. These are areas that had not been envisaged at the time of publication of the first edition of The Macrophage, and are typical of the advances described in the second edition. Overall, I consider the current edition of the book a valuable addition to the literature; a must for all institutional libraries and for laboratories or clinical departments actively engaged in fields involving macrophages, cytokines or immunology.

